# Conformationally Constrained Histidines in the Design of Peptidomimetics: Strategies for the χ-Space Control

**DOI:** 10.3390/ijms12052853

**Published:** 2011-05-03

**Authors:** Azzurra Stefanucci, Francesco Pinnen, Federica Feliciani, Ivana Cacciatore, Gino Lucente, Adriano Mollica

**Affiliations:** 1 Department of Pharmaceutical Sciences, University of Chieti-Pescara “G. d’Annunzio”, Via dei Vestini 31, 66100 Chieti, Italy; 2 Institute of Biomolecular Chemistry (CNR), c/o Department of Chemistry and Pharmaceutical Technologies, “Sapienza”, University of Rome, P.le A. Moro, 00185 Rome, Italy

**Keywords:** amino acids, alkyl substitution, conformation, histidine, stereoselective synthesis, χ-space

## Abstract

A successful design of peptidomimetics must come to terms with χ-space control. The incorporation of χ-space constrained amino acids into bioactive peptides renders the χ^1^ and χ^2^ torsional angles of pharmacophore amino acids critical for activity and selectivity as with other relevant structural features of the template. This review describes histidine analogues characterized by replacement of native α and/or β-hydrogen atoms with alkyl substituents as well as analogues with α, β-didehydro unsaturation or C^α^-C^β^ cyclopropane insertion (ACC derivatives). Attention is also dedicated to the relevant field of β-aminoacid chemistry by describing the synthesis of β^2^- and β^3^-models (β-hHis). Structural modifications leading to cyclic imino derivatives such as spinacine, aza-histidine and analogues with shortening or elongation of the native side chain (nor-histidine and homo-histidine, respectively) are also described. Examples of the use of the described analogues to replace native histidine in bioactive peptides are also given.

## 1. Introduction

Design of peptidomimetics with a specific structure, conformation and topographical properties is a major issue in medicinal chemistry. Peptidic ligands elicit their bioactivity by the interaction of a portion of the three-dimensional (3D) structure with a complementary surface of the acceptor/receptor molecule or molecular complex. The peptide backbone acts as a scaffold which maintains the side chains involved in the interaction in the correct spatial position, providing at the same time the hydrogen bond network necessary to the molecular recognition and binding. The three-dimensional shape (topography) and stereoelectronic properties of the side chain moieties involved in the binding process are critical for the interaction, and provide the necessary complimentary chemical/physical environment for molecular recognition. During the past 30 years much effort has been expended to develop strategies for the design and synthesis of peptides with specific backbone conformation such as α-helix, β-sheet, extended, γ- and β-turn structures. More recently, one of the major efforts has been shifted to the design and synthesis of novel amino acids with conformationally constrained side chains so as to improve the synthesis of highly selective and potent peptide hormone and neurotransmitter analogues.

### 

#### Peptide Dihedral Angles

**Figure 1.**
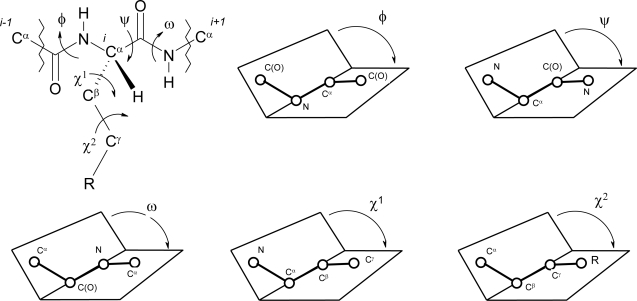
Conformations of peptides: definitions of the Φ, ψ, ω, χ^1^ and χ^2^ torsional angles.

Natural amino acids and peptides exist with a native conformational bias which starts at the backbone level. Torsional (or dihedral) angles commonly used to define the spatial orientation of the backbone peptide bonds and side chains are defined as φ, ψ, ω, and χ as illustrated in Figure 1.

The overall conformation of the peptide molecules is described by the sequence of the backbone torsion angles. The first torsion angle along the backbone is the Φ angle. This is defined as the torsional angle involving the bond sequence *C*(*O*)-*N*-*C*^α^-*C*(*O*) where both *C*(*O*) atoms are carbonyl carbons. The *C*^α^-*C*(*O*)-*N*-*C*^α^ torsional angle is known as the ω angle. Similarly is defined the ψ angle which involves the sequence *N*-*C*^α^-*C*(*O*)-*N*. Finally, the χ torsional angles of the side chain groups of each amino acid are also critical. χ^1^ is defined by the torsional angle *N*-*C*^α^-*C*^ß^-*C*^γ^ and χ^2^ by the torsional angle *C*^α^-*C*^ß^-*C*^γ^-*C*^δ^ and so forth (Figure 1) depending on the structure and extension of the side chain.

The range of the values which Φ, ψ, ω and χ torsion angles can adopt along the backbone and inside each side chain is severely restricted by steric hindrance thus leading to sets of preferred torsional angles.

**Figure 2.**
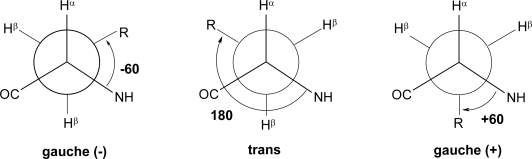
Newman projection of three staggered rotamers in l-amino acids.

The χ^1^ torsional angles are well illustrated by referring to the Newman projection (Figure 2). There is an energy barrier between the discrete angles, with values of +60°, −60°, and 180°having the most favorable energies.

It is important to note that clockwise direction on the Newman projection is defined as a (+) while a counter clockwise direction projection is defined as a (−).

Other angles in the Newman projection are less favorable due to a greater energy to overcome steric hindrance. X-ray crystallography of constrained peptides and calculations clearly show this [1,2]. The torsional angles in the rotamers reported in Figure 2 are the different values of the χ^1^ torsion angle. The ω angle is generally planar and *trans* configured with the *C*^α^ atoms of the two involved residues. Notable exception is that of the X-Pro peptide bond which can be also found as *cis* with deviation from the planarity higher than that of the usual peptide junctions [3,4].

Evaluation of the favored low energy conformations of the Φ and ψ angles was first examined by Ramachandran and co-workers [5], about 30 years ago, and then by many other workers. These studies show that only certain regions of the Φ, ψ space (often referred to as Ramachandran space) are actually accessible to l-amino acids. Interestingly these regions correspond to the classical secondary structures of peptides and proteins (α-helix, β-sheet, extended, *etc.*). An equally critical area, though much less explored and relevant to the design of new structures, is that of the 3D shape of amino acid side chains.

As illustrated in Figure 2, each χ^1^ torsional angle (and some of the χ^2^, χ^3^, *etc.* subsequent torsional angles along the chains) can adopt three low energy staggered rotameric conformations: *gauche*(−), *gauche*(*+*), and *trans*. The corresponding energy conformation is not expected to be high (about 1–2 kcal/mole for most of the simple amino acid residues).

Yet the orientation of the side chain of l-amino acid residues, with respect to the peptide or protein backbone, will be dramatically different in each of the three cases: for *gauche*(−) χ^1^: orientation towards the *N*-terminus; for *trans*: orientation towards the *C*-terminus; for *gauche*(*+*): orientation over the backbone. In molecular recognition processes, the side chains will adopt generally one of these three possible conformations, with preference for the *gauche*(−) > *trans* > *gauche*(+) for most natural l-amino acid residues. Clearly, the side chain conformations are critical to the molecular recognition and information transfer processes involving ligand-receptor/acceptor interactions [6,7].

## 2. Background

The incorporation of χ-space constrained amino acids into bioactive peptides renders the χ^1^ and χ^2^ torsional angles of pharmacophore amino acids critical for activity and selectivity as with other relevant structural features of the template.

A successful design of peptidomimetics in χ-space will depend upon several conditions: (1) side chains should adopt the preferred conformation found in natural amino acids; (2) the constraint should be compatible with backbone conformations; and (3) the constraint should be compatible with efficient binding kinetics. These are critical issues because the constrained amino acid residues must not significantly interfere with the conformation of the constrained template, with binding of the ligand to its receptor, or with the dynamic processes and conformational changes that accompany ligand-receptor interaction and information transduction [2].

**Figure 3.**
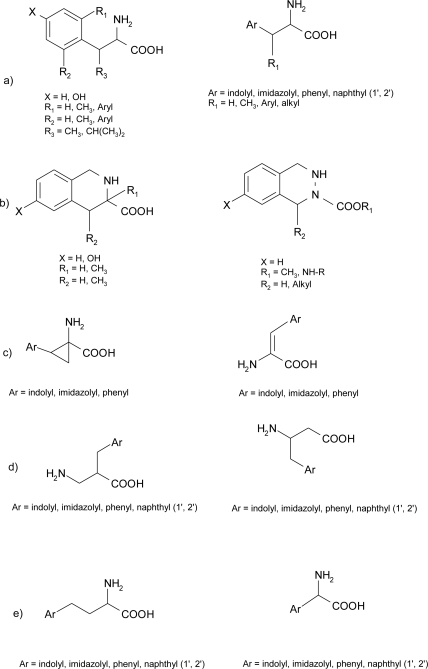
General structure of some χ-constrained aromatic amino acids: (**a**) β and side chain alkyl substituted amino acids; (**b**) imino acids; (**c**) α-β dehydro and α-amino cyclopropanecarboxylic acid derivatives (ACC); (**d**) β^2^/β^3^-homo-amino acids; (**e**) homo and nor-amino acids.

During the design based on the topographical χ space control, authors concentrated their major efforts on aromatic amino acids. All peptide hormones and neurotransmitters have in fact one or more aromatic amino acid residues as key pharmacophore. Different structural features, chosen in order to constrain the χ space of aromatic amino acids, are shown in Figure 3.

Histidine (His) is an important amino acid critically involved in receptor recognition and biological activity of many peptides and proteins [8].

The peculiar chemical properties of the imidazole ring are responsible for this. Histidine is often strategically located in a variety of biologically active peptides such as angiotensin [9], luteinizing hormone releasing hormone (LHRH) [10], liver cell growth factor [11], thyrotropin releasing hormone (TRH) and is preferential binding site for transition metal ions [12].

Despite the great attention dedicated to the structural modifications performed on phenylalanine, tyrosine and tryptophan aromatic residues and to the conformational and biochemical consequences of the incorporation of the obtained synthetic analogues into peptide backbones, the histidine residue has been only marginally treated. The presence of the basic and nucleophilic imidazole ring, which induces racemization processes and requires almost invariably specific temporary protection, renders the synthetic strategies more laborious than those encountered with any other aromatic amino acid. Here we summarize some relevant papers performed in the field of histidine analogues with focus on constrained models, characterized by the presence of α- and/or β-substituents, capable of limiting the χ-space available to the imidazole side chain. Results concerning other relevant analogues, such as those pertaining to the relevant field of β-amino acids are also reviewed.

## 3. Nomenclature

### 

#### Histidine Derivatives

Since the nomenclature of imidazole ring and histidine have been subjected to several changes in the years, data reported here adopt the following nomenclature: the nitrogen atoms of imidazole ring are denoted by *pros* (π) and *tele* (τ) to show their position relative to the side chain (*near* and *far*, respectively).

**Figure 4.**
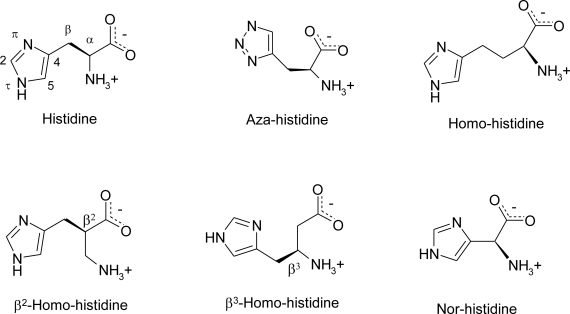
Adopted notations for histidine and its analogues.

The carbon atom between the two ring nitrogen atoms is numbered 2 (as in imidazole), and the carbon atom next to the τ nitrogen is numbered 5. The carbon atoms of the aliphatic chain are designated α and β [13] (Figure 4).

## 4. Chemistry

### 4.1. α-Alkyl Substitution

The strategy based on replacement of α and β hydrogen atoms of histidine with alkyl groups started when topographical considerations, relevant to the stereochemical features required for receptor recognition and signal transduction, were still not well established.

It has been demonstrated that α substitution poorly affects in general the χ^1^ torsional angles. The more effective changes are found on the backbone and these analogues are in fact considered α-helix and β-turn inducers [14].

**Scheme 1.**
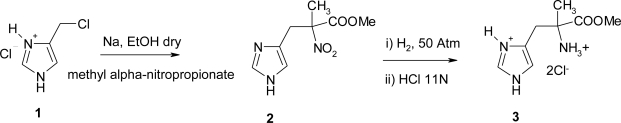
Synthetic procedure for α-methyl histidine [15].

An early synthetic method (reported by Robinson and Shepherd [15]) for the α-substituted product **3** is the coupling reaction between methyl α-nitropropionate and chloromethylimidazole **1** to give **2** followed by reduction of the nitro group (Scheme 1).

**Scheme 2.**
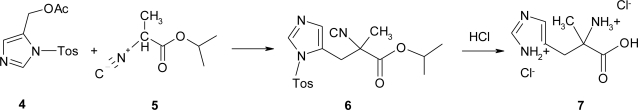
Synthetic procedure for α-methyl histidine [16].

Successively (1974), Suzuki *et al*., found that *N*-tosyl-acetoxy-methylimidazole [16] **4**, easily prepared from hydroxymethylimidazole [17], is a useful intermediate for the introduction of imidazole ring in the amino acid skeleton. Accordingly, α-methylhystidine was synthesized by reaction of α-isocyanoproprionate **5** with *N*-tosyl-acetoxymethylimidazole **4**, as shown in Scheme 2.

The coupling product **6** was obtained in 67% yield and was subsequently hydrolyzed with HCl to give α-methylhistidine dihydrochloride **7** in 80% yield.

**Scheme 3.**
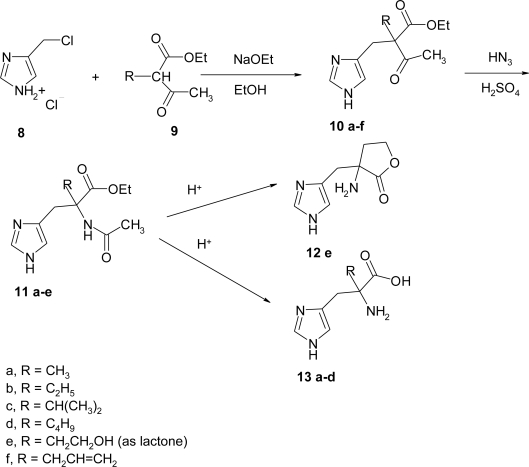
Synthetic procedure for α-alkyl histidine [18].

Two years later DeGraw *et al*. used the route outlined in Scheme 3 to synthesize α-substituted histidines. 4-Chloromethylimidazole hydrochloride **8** [18], was added to a solution of an appropriate 2-alkyl acetoacetate **9a**–**d**,**f** or 2-acetylbutyrolactone **9e** in ethanol solution containing sodium ethoxide to afford the 2-alkyl 2-(4-imidazolylmethyl)acetoacetate **10**. When the keto ester **10** was allowed to react with a slight excess of hydrazoic acid in sulfuric acid solution, the *N*-acetylhistidine esters **11a**–**e** were obtained in 50–70% yields. Under the acidic conditions employed, the α-allyl-analogue **10f** was apparently subjected to additional attack on the vinyl moiety, leading to an unidentified product rather than the anticipated product **11f**. Schmidt [19] had qualitatively shown that in β-keto esters the HN_3_ reagent afforded selective attack on the ketone carbonyl with subsequent rearrangement occurring at the α-carbon. Synthesis of the histidines **12** and **13** were completed by acid hydrolysis of the amide and ester functions. This route seems to have been little used in the synthesis of α-substituted amino acids, but especially valuable in the histidine series.

**Scheme 4.**
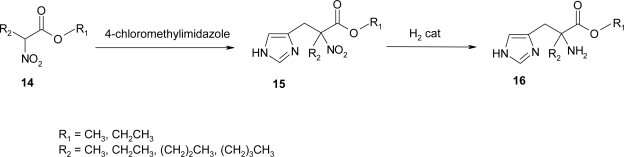
Synthetic procedure for α-alkyl histidine [15,20].

An alternative synthesis (Scheme 4) of α-substituted histidine was proposed by Kelley in 1977. The synthesis was modeled after the work of Robinson and Shepherd [15] and Sletzinger and Pfister [20], as outlined in Scheme 4.

The appropriate α-nitro ester **14** [21], (Scheme 4) was alkylated with 4-chloromethylimidazole [15], in methanol or dimethylformamide. In the case of the α-phenyl analogue **14** (R_1_ = CH_2_CH_3_; R_2_ = C_6_H_5_) hexamethylphosphoramide was required to obtain the product, even if the yield remained low. Reduction of the nitro esters **15** was best accomplished by catalytic hydrogenation to give the desired product **16**. Although the reduction required a high catalyst to substrate ratio and extended reaction times, in all but one case the products were obtained in high yield and purity.

**Scheme 5.**
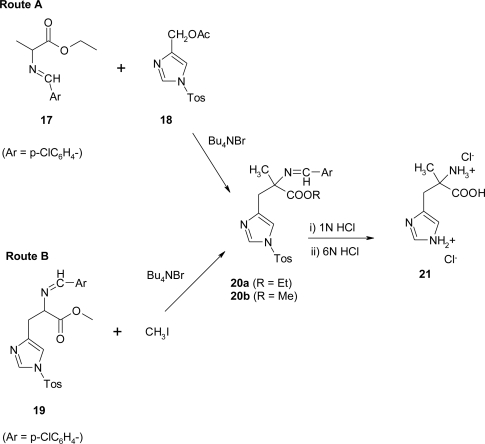
Synthetic procedure for α-methyl histidine [22].

In 1989, O’Donnell *et al*. [22] synthesized α-methyl histidine by two complementary routes using a catalytic phase-transfer (PTC) alkylation procedure (Scheme 5).

The first route involves benzylation of the Schiff base ester **17**, prepared in 90% yield from alanine ethyl ester hydrochloride and *p*-chlorobenzaldehyde, with the electrophile **18** [23]. In the second approach the appropriately protected histidine substrate **19** is α-methylated by using the PTC method. The intermediate **19** is easily prepared in 83% yield in one pot procedure from histidine methyl ester dihydrochloride by condensation of the free amino ester (generated *in-situ*) with *p*-chlorobenzaldehyde followed by protection of the imidazole nitrogen with tosylchloride/triethylamine. Alkylation of **17** or **19** was accomplished by catalytic solid-liquid phase transfer alkylation using powdered potassium hydroxide in acetonitrile at 15–20 °C with 10% Bu_4_NBr as the phase-transfer catalyst. The crude alkylated derivatives **20** (**20a**, R = Et, 47% crude; **20b**, R = Me, 70% crude), obtained as oil, were hydrolyzed directly in a two-steps sequence. The product α-methyl histidine was obtained as its dihydrochloride **21** in overall yields of 27% and 41% from **17** and **19**, respectively.

### 4.2. β-Alkyl Substitutions

**Scheme 6.**
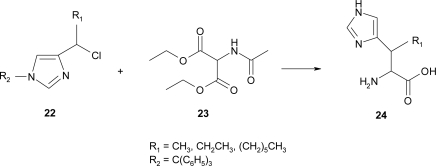
Synthetic procedure for β-alkyl histidine [24].

A synthetic route for β-alkylhistidines **24** was reported by Kelley [24] and co-workers as a modification of Albertson’s classic synthesis of histidine [25], by using the appropriate 1-(4-imidazolyl)alkyl chloride **22** and the diethyl acetamidomalonate **23** (Scheme 6).

The synthetic route afforded the desired amino acids as diastereoisomeric mixtures. No attempt was made to separate the diastereoisomers.

**Scheme 7.**
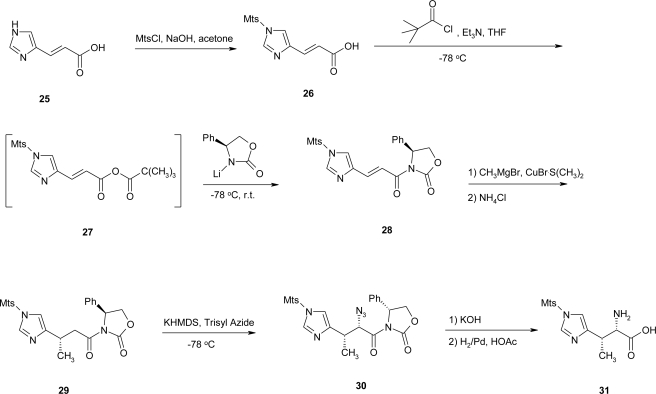
Synthesis of (2S,3S)-β-methylhistidine [8].

A successful asymmetric synthesis of (2S,3S)-β-methylhistidine **31**, by using 2-mesitylenesulfonyl (Mts-) as a protecting group at τ-*N* in the imidazole ring was reported by Hruby *et al*. [8] (Scheme 7).

The synthesis started from the commercially available urocanic acid **25**. This was treated with 2-mesitylensulfonylchloride MtsCl in sodium hydroxide solution to furnish the derivative **26** τ-N protected at the imidazole ring. The protected urocanic acid was coupled by mixed anhydride method **27** to an optically pure chiral auxiliary according to a reported procedure [26] to give **28**. The subsequent Michael addition and azide formation followed a highly enantioselective route (>99% as determined by ^1^H NMR). Hydrolysis of the chiral auxiliary **29** to compound **30** led to the removal of the Mts-protecting group. The final unprotected β-methylhistidine **31** was isolated, purified by ion-exchange column (Dowex 50X2-100) and recrystallized from water/ethanol [27]. According to the ^1^H NMR spectra some epimerization was observed in the last two steps; thus the Mts- or another protecting group should be reintroduced for this use in peptide chemistry. Although the free imidazole group can apparently catalyze the epimerization process, the adoption of the Mts protection allowed the first successful asymmetric synthesis of a (2S,3S)-β-methylhistidine derivative.

**Scheme 8.**
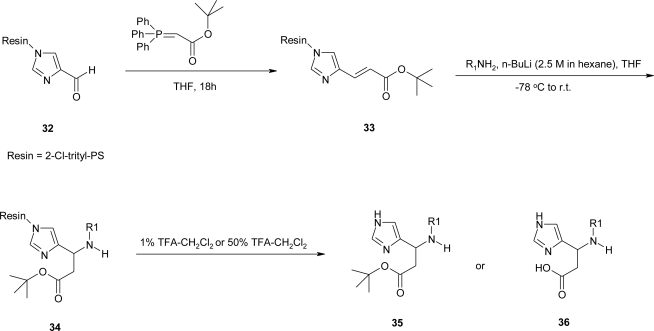
Aza-Michael reaction toward synthesis of β-amino acids on solid support [28].

Recently, Saha *et al*. [28] synthesized β-amino acids based libraries on solid support, ancoring 4-formyl imidazole to 2% cross-linked PS-resin using the convenient 2-Cl trityl linker. Horner-Emmons condensation was conducted on resin bound aldehyde with an excess of tert-butylcarboxy triethyl phosphonoacetate in THF **32**. Lithium amides have been utilized in conjugate Aza-Michael additions to α,β-unsatured systems for the generation of protected chiral β-amino acids **34** [29]. The amides were generated by pre-treating several amines with n-BuLi in THF at −78 °C. The resulting lithium amides were added via cannula under N_2_ to a pre-swelled suspension of resin **33** in THF, maintained at −78 °C. Then, the mixture was warmed to room temperature followed by work up procedure. Cleavage after Aza-Michael addition, under mild-TFA conditions produced the tert-butyl ester products **35**. Cleavage with 25–50% TFA-CH_2_Cl_2_ gave the deprotected β-amino acid products **36** (Scheme 8).

### 4.3. β,β–Dimethyl Substitution

In 1976, De Graw *et al*. [30] prepared a series of substituted histidines and homo-histidines and evaluated the obtained compounds as inhibitors of specific histidine decarboxylase obtained from rat pyloric stomach.

**Scheme 9.**
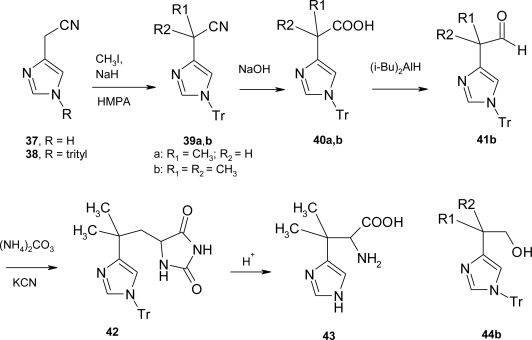
Synthesis of β,β-dimethyl histidine [30].

β,β-dimethyl histidine **43** was prepared by the procedure shown in Scheme 9; 4-cyanomethylimidazole **37** was conveniently obtained in 70% yield from histidine by decarboxylation in sodium hypochloride solution [31].

Treatment of **37** with bis(trimethylsilyl)acetamide afforded an *N*-Me_3_Si intermediate which, when allowed to react with trityl chloride, gave the *N*-trityl-4-cyanomethylimidazole isomer **38** in 77% yield. Although shown as the 1-*N*-trityl derivative **39**, the true position (*N*^τ^ or *N*^τ^) of tritylation was not established. The blocked nitrile **38** was converted to the anion by treatment with sodium hydride in hexamethylphosphoramide (HMPA) solution. Subsequent reaction with methyl iodide at room temperature afforded a mixture of methylation products containing approximately 77% of the monomethylnitrile **39a**. The dimethylnitrile **39b** was obtained by exhaustive re-treatments to ensure complete methylation. Hydrolysis of the nitriles was effected by sodium hydroxide in hot 90% 2-methoxy ethanol to afford the carboxylic acids **40a** and **40b** in 84% and 86% yields, respectively. Reduction of the dimethyl acid **40b** with diisobutylaluminum hydride in toluene gave a mixture containing about 75% of the aldehyde **41b** as shown by NMR analysis. The crude product was chromatographed to remove unreacted acid **40b** and to effect a separation of the aldehyde from an undesired product regarded as the alcohol **44b**. The crystalline aldehyde **41** was allowed to react with KCN/(NH_4_)_2_CO_3_ at 110 °C in a bomb tube [32], to yield the hydantoin **42** as a crystalline product. Acid hydrolysis of **42** yielded the β,β-dimethyl histidine **43**.

### 4.4. Constrained “Imino Acids” 1,2,3,4-Tetrahydroquinoline Derivatives (Spinacines)

The most important procedure to synthesize cyclic analogues of histidine uses the Pictet-Spengler reaction [33,34] or modifications thereof, by cyclocondensation of the amino acid His with formaldehyde in the presence of concentrated hydrochloride acid. In general these reactions proceed in good yield (70–97%) and enantiomerically pure amino acids can be obtained from enantiomerically pure precursors. If partial racemization [35] occurs fractional crystallization is necessary to obtain the desidered amino acid (yields: 90–99%) [36,37].

Among the variety of histidine analogs which provide a conformational restriction of the peptide backbone and/or of the lateral chain, the most extensively employed in structure-activity and metal-ion complexation studies have been the *N^α^*-, *N^τ^*- and *N^π^*-methyl histidine derivatives [38]. More recently, the (4,5,6,7-tetrahydro-1*H*-imidazo[4,5-c]pyridine-6-carboxylic acid) (Spinacine, Spi) has also been studied [39,40].

**Scheme 10.**
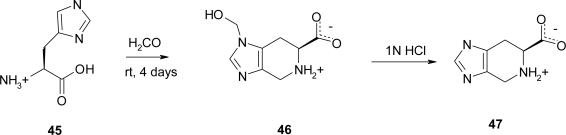
Synthesis of spinacine (Spi) and hydroxymethyl-spinacine Spi(π-OMe) [43].

Reaction of L-His and formaldehyde to give Spi **47** was first reported by Wellisch in 1913 [41], but only in 1991 Klutchko *et al*. [42] described the synthesis via Pictet-Spengler reaction on *N^π^*-substituted histidines, of a large number of Spi derivatives including amide, ester, 5-alkyl and acyl, and regiospecific *N^π^-*akyl and aralkyl derivatives.

l-Spi has been also synthesized under different experimental conditions which gave, as an intermediate, its *N^π^*-hydroxymethyl derivative Spi(π-MeOH) [43] **46**. In this case, the starting l-His in 0.1 M sodium phosphate buffer (pH 7.0) was reacted with a 30-fold molar excess of formaldehyde at room temperature for 4 days; the precipitate was collected by filtration, washed with cold water and dried. The product, separated from the reaction medium as crystal suitable for X-ray diffraction, was obtained in 93% yield (Scheme 10). The obtained Spi(π-MeOH) **46** was dissolved in 1 M HCl and the solution concentrated at reduced pressure. The residue **45** was dissolved in water and the pH adjusted to 3.5. Absolute ethanol was added, obtaining crystals which were collected by filtration, washed with ethanol and dried (95% yield).

**Scheme 11.**
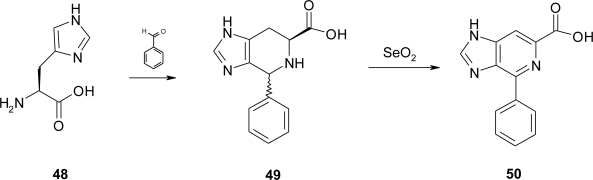
Synthesis of spinacine and derivatives [44].

An interesting use of the amino acid spinacine is reported by Guzman *et al*. [44] in their synthesis of *cis/trans* imidazopyridines spinacine derivatives (Scheme 11):

In particular, the 1-phenyl derivative **50** was synthesized by the method of Wille [45] through the base-catalyzed Pictet-Spengler reaction of histidine **48** with benzaldehyde. Aromatization of the tetrahydroimidazopyridine derivative **49** with selenium dioxide readily afforded **50** as free base.

#### X-ray Studies on Spinacine Derivatives

Two tautomeric forms can be predicted for the amino acid spinacine, depending on the position of the hydrogen on the nitrogen atoms of the imidazole ring.

**Figure 5.**
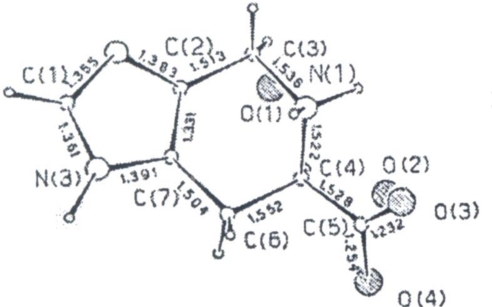
An ORTEP view of Spi [46].

X-ray studies reported by Andreetti *et al*. [46], showed that the crystals correspond to the tautomer having the *N*(3) atom protonated as shown in Figure 5, in which the bond distances are also reported. Thus, the compound corresponds to the amphionic form of the 4,5,6,7-tetrahydro-1H-imidazo[4,5-c]pyridine-6-carboxylic acid. Condensation of imidazole with tetrahydropyridine removes the π electron delocalisation on the imidazole and produces a double bond localized on *C*(2)-*C*(7) [*C*(2)-*C*(7) = 1.331(10) Å]. The other bond distances and angles are in agreement with the hybridisation state of the atoms. The imidazole ring and the *C*(6) and *C*(3) atoms lie on a plane whereas *C*(4) and *N*(1) are out of that plane by 0.208 and −0.568 Å respectively; this as a consequence of the sp^3^ character of the four atoms of the six-membered ring. The torsional angles around the *N*(1)-*C*(3) and *C*(4)-*C*(6) are −25.6° and 30.8° respectively. The carboxyl group is equatorial and orientated in such a way that one oxygen points to the NH_2_^+^ group to compensate the opposite electrical charge.

**Figure 6.**
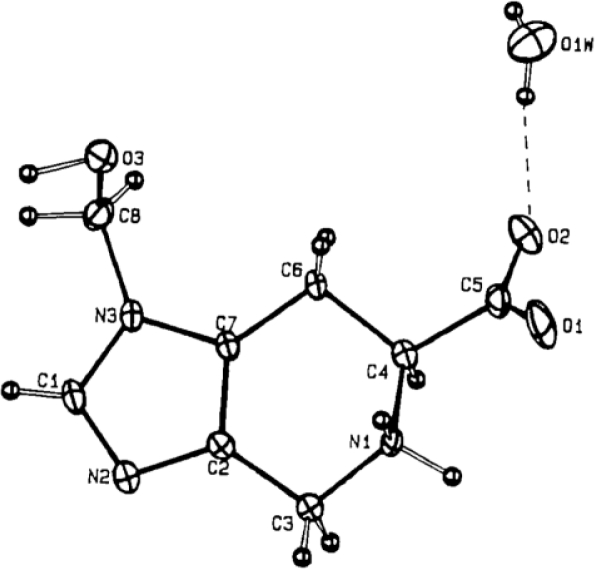
An ORTEP view of Spi(πMeOH)·H_2_O showing thermal ellipsoid at 30% probability [43].

Solid state structure of spinacine analogues have been recently reported by Bertolasi *et al*. [43] (Figure 6) and one of these is that Spi(πMeOH) **46** which crystallizes with a water molecule and its structure displays, like that of Spi, a zwitterionic character. The carboxylate group is situated in equatorial position and is rotated by about 10° around the *N*1-*C*4 bond, the torsion angles *O*1-*C*5-*C*4-*N*1 and *O*2-*C*5-*C*4-*N*1 being −10.2(2)° and 170.3(2)°, respectively. This conformation, observed also in the structure of Spi and other amino acids [46], is favored by a short electrostatic interaction of 2.618(2) Å between the negative charged *O*2 atom and the positive *N*1. Bond distances and angles are within the normal ranges; in particular, comparing the present structure with those of imidazole [47], and l-His [48], it can be observed that the hydroxymethyl substituent at *N*3 and the condensation with tetrahydropyridine do not produce significant variations in the imidazole geometry. The tetrahydropyridine ring *C*2-*C*7-*C*6-*C*4-*N*1-*C*3 adopts a half-chair conformation ^5^H_4_ with puckering parameters Q_T_ = 0.483(1) Å, φ = −145.6(3)°, θ = 48.0(2)° and ΔC_2_(C2–C7) = 0.0182(7) Å [49,50].

### 4.5. 1-Amino-2-(4-imidazolyl)cyclopropanecarboxylic Acid Derivatives (ACC)

The most general route to His derivatives containing a cyclopropane ring between the *C^α^* and the *C*^β^ carbon atoms (ACC derivatives) involves cyclopropanation of 2-aryl-4-benzylideneoxazolones, formed by 1,3-dipolar cycloaddition of diazomethane followed by thermolysis of the intermediate pyrazoline. The cyclopropanation reaction works moderately well and the subsequent chemical transformations provide the amino acids in low to moderate overall yield (8–38%) [51].

**Scheme 12.**
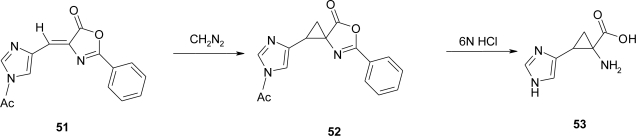
Synthesis of ACC derivative [52].

An attractive pathway to ACC **53** appears the addition of diazomethane to 4-[(1-acetyl-4-imidazolyl)methyl-ene]-2-phenyl-2-oxazolin-5-one **51** followed by hydrolytic cleavage of the oxazolone ring **52** [52] (Scheme 12).

The choice of this route follows the findings of Awad *et al*. [53] and Mustafa *et al*. [54], according to which the carbon-to-carbon double bonds, exocyclic to certain hetero rings, including oxazolones, react with diazomethane to give cyclopropane derivatives. Moreover, Awad *et al*. [53], have cleaved the azlactone 1,5-diphenyl-6-oxa-4-azaspiro-[2,4]hept-4-en-7-one to 1-benzamido-2-phenylcyclopropanecarboxylic acid thus showing that the *N*-benzoyl group could be hydrolyzed under conditions which would not affect the cyclopropane ring.

### 4.6. α,β-Dehydro Amino Acids

In 1990 Easton and co-workers reported a method for the diastereoselective conversion of amino acids to their β-hydroxy derivatives by direct side chain bromination of the amino acid derivatives with NBS, followed by treatment with silver nitrate in aqueous acetone [55–57].

The side chain bromination requires an *N*-protecting group, such as phthaloyl or trifluoromethanesulfonyl, in order to deactivate the α-position toward hydrogen atom abstraction [58].

**Scheme 13.**
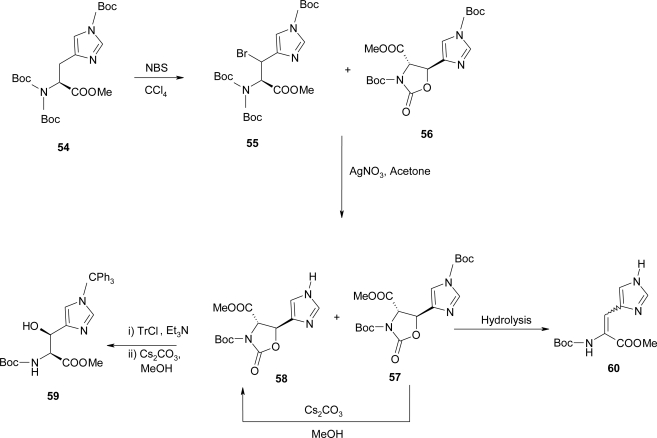
Synthesis of α,β-dehydro-histidine [59].

Crich *et al*. [59] reported the use of *N,N*-di-tert-butoxycarbonyl protected amino acids in Easton’s protocol and the advantages that this strategy affords to the synthesis of α,β-dehydrohistidine and β-hydroxyhistidine (Scheme 13).

The radical bromination of **54** [60] provided the threo bromide **55** and the trans oxazolidinone **56**. This mixture was subsequently treated with silver nitrate in acetone to afford the *trans* oxazolidinones **57** and **58**, which differ by the presence of τ *t*-butoxycarbonyl group, in 62% yield. Attempted hydrolysis of 57 or 58 under a wide variety of conditions produced the α,β-dehydrohistidine derivative **60**. The free imidazole nitrogen is responsible for the elimination reaction thus, imidazole protecting group, stable under mild basic conditions, would solve the problem. Product **58** was also reacted with trityl chloride and triethylamine in dichloromethane and, after removal of the excess trityl chloride, the reaction mixture was treated with catalytic cesium carbonate in methanol leading directly to the formation of the desired (2S,3S) β-hydroxyhistidine derivative **59** in 74% yield (Scheme 13).

**Scheme 14.**
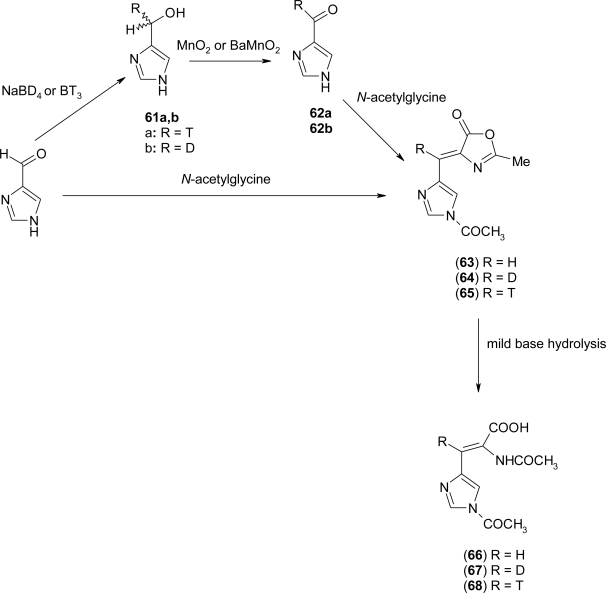
Synthesis of α,β-dehydro-histidine [61].

In 1980 Battersby *et al*. [61] realized a stereoselective synthesis of (αS, βS)-[β-^3^H_1_]histidine and (αS, βR)-[β-^3^H_1_]histidine, obtaining as intermediate, the 2-acetamido-3-[imidazol-4(5)yl-acrylic]acid (Scheme 14). In the first approach, NaBD_4_ or BT_3_ were added to a solution of 4(5)-formylimidazole to obtain compound **61a** or **61b**. These were then oxidized by MnO_2_ or BaMnO_2_ to the aldehyde **62a** or **62b**. Condensation of the aldehyde with *N*-acetylglycine in acetic anhydride afforded the oxazolinone **64** and **65** which were converted into the required acrylic acid **67** and **68** by mild basic hydrolysis. In the second way, 4(5)-formylimidazole was directly condensed with *N*-acetylglycine to obtain compound **63**. Then **63** was worked up as above to give the imidazolylacrylic acid **66**.

Cativiela *et al*. [62] published the synthesis, based on the method of Battersby [61], of α,β-dehydro-histidine with Z geometry around the double bond (Δ^Z^-His). The synthesis starts from 5-formylimidazole in Ac_2_O/AcONa which reacts with hippuric acid to give the intermediate azlactone; subsequent hydrolysis with sodium carbonate in water gave the desired Z configured compound.

**Scheme 15.**
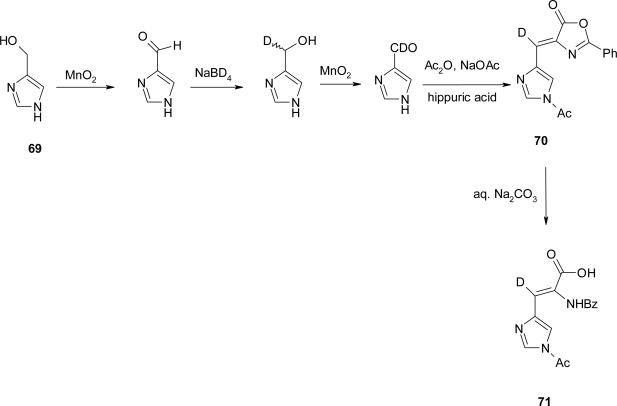
Synthesis of (Z)-α,β-dehydro-histidine (Δ^Z^ His) [63].

Recently, an example of azlactonization of α,β-dehydro-histidine (**71**) starting from 5-hydroxymethyl-imidazole (**69**) was reported by Parker *et al.* [63]; as illustrated in Scheme 15, (2S, 3S)-[3-^2^H]histidinol, was synthesized by a stereochemically unambiguous route.

**Figure 7.**
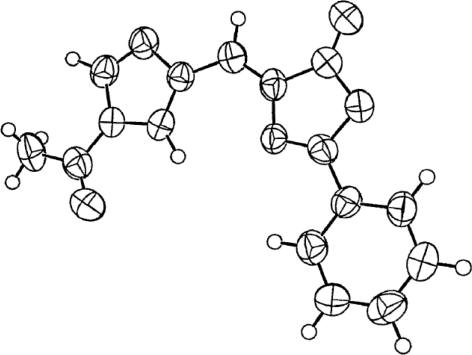
An ORTEP view of azlactone **70** [63].

Azlactone **70** was crystallized and its structure determined by X-ray crystallography. The ORTEP drawing of **70** (Figure 7) clearly indicates that the exocyclic double bond has the Z configuration, as shown in Scheme 15.

X-ray crystal structure of azlactone **70** is illustrated in Figure 7.

**Scheme 16.**
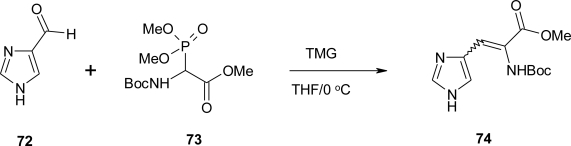
Synthesis of α,β-dehydro-histidine [64].

A Japanese patent [64] reported the synthesis of a series of new imidazole derivatives (Scheme 16).

The 5-formylimidazole **72** is treated with tert-butoxycarbonylamino-(dimethoxy-phosporyl)-acetic-acid methyl ester **73** in THF in the presence of 1,1,3,3-tetramethylguanidine (TMG) at 0 °C. The product (**74**) of the condensation reaction is E/Z diastereoisomeric mixture.

### 4.7. Homo-Histidine

**Figure 8.**
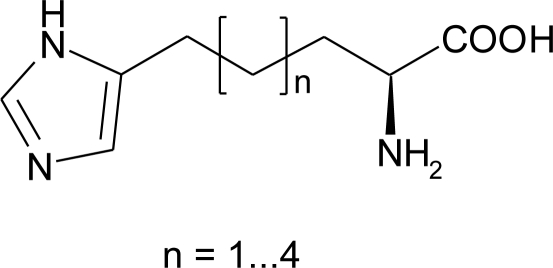
Series of l-homo-histidines.

**Scheme 17.**
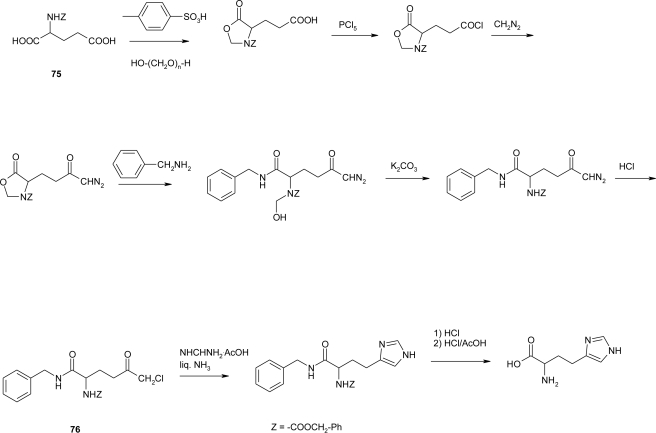
Synthesis of l-homo-histidine [66].

The ten-steps synthesis of l-homo-histidine (Figure 8) by Bloemhoff and Kerling [65] using *N*-benzyloxycarbonyl-l-glutamic acid **75** as starting material, was revisited by Altman *et al*. [66] (Scheme 17).

The imidazole ring was built from the gamma-carboxyl group while the α-function was protected. The protecting group was removed in the last step. The critical step in this procedure involves imidazole ring closure with ammonia and formaldehyde forming copper salt. The reaction appears to be the most tedious step and does not exceed 50% yield. By submitting chloromethyl chetone **76** to cyclization conditions with formamidine acetate in ammonia the yield was greatly improved (71%), leading to the overall yield of 40%.

**Scheme 18.**
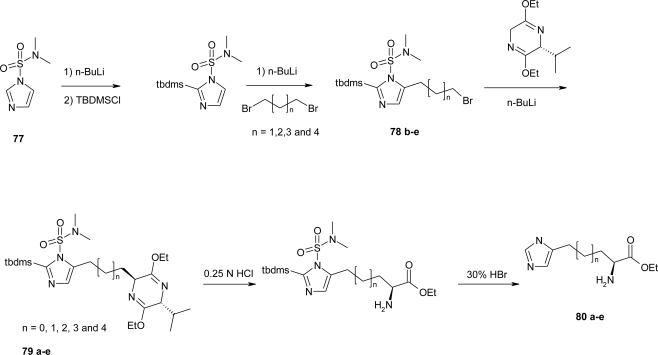
Synthesis of l-homo-histidines [67].

This method is not applicable to the preparation of a series of imidazole-containig amino acid derivatives by Silverman *et al*. [67] thus, a new enantioselective general procedure leading to **80a**–**e** (Scheme 18) was devised by utilizing the reaction of various imidazolyl alkyl bromides **78b**–**e** with chiral lithiated bislactim ether **79** as the key step.

1-(*N,N-*dimethylsulfamoyl)imidazole **77** was protected with a tert-butyldimethylsilyl group (TBDMS) by sequential treatment with *n*-butylithium followed by tert-butyldimethylsilyl chloride. The lithium salt of the 1,2-diprotected imidazole was allowed to react with a series of dibromoalkanes to afford bromoalkyl substituted imidazoles **78b**–**e**. Carbon-carbon bond formation between the alkyl bromides and lithiated bislactim ether proceeded in good yields. The homologated bislactim ether products **79a**–**e** were hydrolyzed with 0.25 N HCl to the corresponding amino acid ethyl ester. Under these reaction conditions the TBDMS protecting group of the imidazole was cleaved. Removal of the sulfamoyl group requires relatively vigorous conditions (refluxing 30% HBr for 4 h), with consequent ethyl esters hydrolysis and formation of the desired amino acids **80b**–**e**. The bromoethyl imidazole analogue **78a** was not prepared by the route used for the other analogues, because the dibromoethane underwent elimination of HBr in the presence of *n*-butyllithium, and not a trace of the substitution product **79a** was detected.

**Scheme 19.**
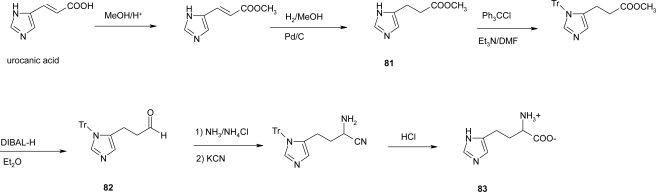
Synthesis of racemic-homo-histidine starting from urocanic acid [68].

A preparation of racemic homo-histidine from the readily available urocanic acid has been reported by Pirrung *et al*. [68] (Scheme 19).

Urocanic acid was esterified and hydrogenated to give compound **81**. As reported by Browne [69], the tritylation significantly improves its solubility in ethereal solvents, permitting DIBAL-H reduction in quantity to the aldehyde **82**. Strecker reaction gives an aminonitrile whose hydrolysis, accompanied by the trityl group removal, produces homo-histidine **83** in 73% overall yield.

A patent reported the synthesis of homo-histidine [70], useful for treating renin associated hypertension.

### 4.8. Nor-Histidine

**Scheme 20.**
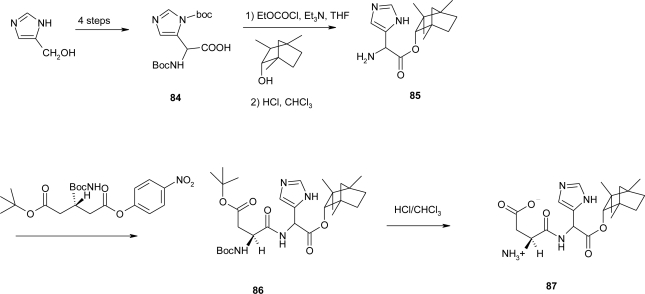
Synthesis of imidazolylglycine sweetener containing nor-histidine [72].

The accidental discovery of sweet dipeptide derivative l-aspartyl-l-phenylalanine methyl ester known as aspartame, was published several years ago [71]. Since then, a variety of analogues have been prepared by different research groups seeking more stable and more potent dipeptides. The synthesis of imidazolylglycine [72] sweetener containing nor-histidine is given in Scheme 20:

The protected *N^α^*,*N^π^*-diBoc-imidazolylglycine derivative **84** was prepared by modification of a published procedure for the *N*^α^-Boc derivative [73,74]. Mixed anhydride coupling with (−)-α-fenchol gave Boc-amino ester in low yield (25–30%). A major side product was the ethyl ester formed by the reaction of ethanol released from the mixed anhydride upon reaction with fenchol. This severe limitation in the use of mixed anhydrides for ester formation is not a problem in amide formation where the nucleophilicity of the amine exceeds that of the ethanol released. Alternative coupling procedures also gave low yields. Acid hydrolysis gave amine **85**. Coupling with *N-(t*ert-butoxycarbony1)-β-tert-butyl-l-aspartic acid p-nitrophenyl ester to **86** followed by acid hydrolysis gave sweetener **87.**

**Figure 9.**
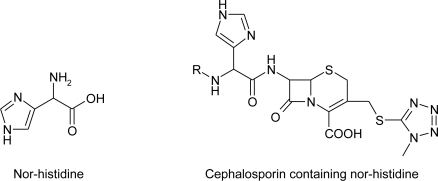
Structure of nor-histidine and cephalosporine containing nor-histidine (Reference 75).

The importance of nor-histidine (Figure 9) as residue in biologically active compounds is shown in the study of Tagawa *et al*. [75].

**Scheme 21.**
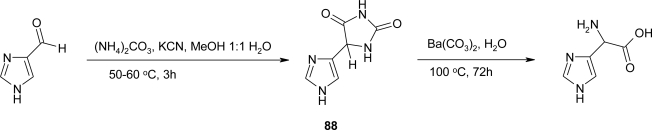
Synthesis of imidazolylglycine via the corresponding hydantoin intermediate [76].

Recently, a panel of amino acid analogs and conformationally-restricted amino acids bearing a sulfonic acid were synthesized and tested for their ability to preferentially inhibit the obligate cysteine glutamate transporter system x_c_ *versus* the vesicular glutamate transporter (VGLUT) [76]; The target conformationally-restricted amino acids were synthesized as shown in Schemes 21; imidazolylglycine was synthesized via hydrolysis of the corresponding hydantoin intermediate **88** [77–79].

### 4.9. β^2^-Homo-Histidine

**Scheme 22.**
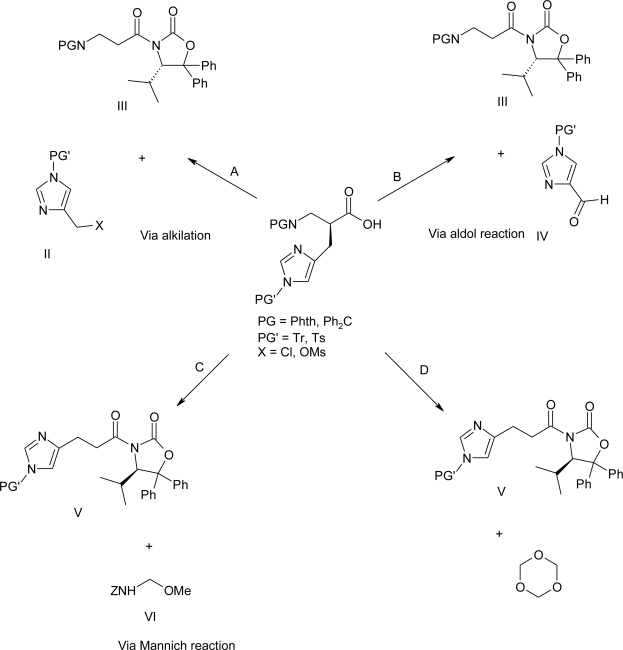
Retrosynthetic analysis for the preparation of the Fmoc-β^2^hHis(Tr)-OH via alkylation, Mannich-Type reaction and aldol addition of chiral acyloxazolidinones [80].

Due to the nucleophilic character of *N*-atom on the (1*H*-imidazolyl)methyl side chain of histidine, several undesired by-products can be formed during the synthesis of β-homo-histidines. Indeed, many routes have been tested, and most were not successful. (Scheme 22) [80].

**Scheme 23.**
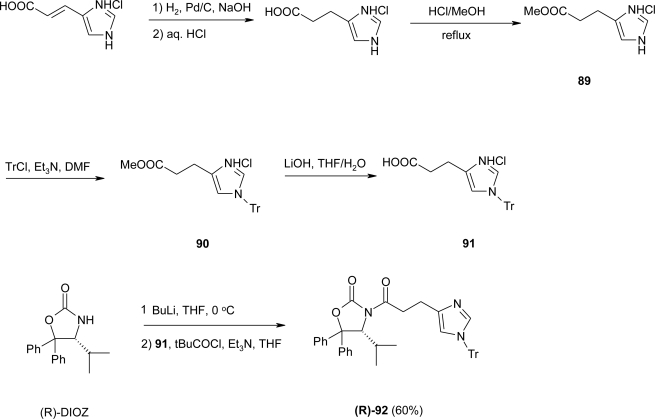
Preparation of the acyloxazolidinone **(R)-92** starting from urocanic acid [80].

Initial attempts of diastereoselective alkylation (*cf.* A in Scheme 22) of homo-glycine derived acyloxazolidinones III (PG = Phth; PG = Ph_2_C) with (1*H*-imidazol-4-yl)methyl derivatives II (PG’ = Trt, X = Cl; PG’ = Ts, X = MsO) were ineffective due to the low reactivity. In a second approach (*cf*. B in Scheme 22), the aldol addition of the oxazolidinone derivatives III with aldehydes IV (PG’ = Tr; PG’ = Ts), followed by deoxygenation under Barton-McCombie conditions, resulted in the isolation of degradation products caused by retro-aldol reactions, occurring during the oxygenation step. Thus, the amidomethylation reaction via Ti-enolates was attempted (*cf.* C in Scheme 22): treatment of the acyloxazolidinone V (PG’ = Tr) with the electrophile VI resulted in a complex mixture of inseparable products; although the desired compounds had been formed, long reaction times were required for good conversion, causing the partial cleavage of the trityl protecting group. Seebach *et al*. envisaged the use of a more reactive electrophile, for instance, 1,3,5-trioxane (*cf*. D in Scheme 22), with subsequent OH/NH_2_ replacement. This route led eventually to the synthesis of the desired β^2^-homo-histidine derivatives for solid phase syntheses. For the preparation of the acyloxazolidinone **92**, 1*H*-imidazole-4-acrylic acid (urocanic acid) was selected as the starting material. Hydrogenation of the urocanic acid, followed by esterification under acid conditions, gave the methyl ester **89**, which was trityl protected to give crude **90**. Product **90** was saponified to afford the acid **91** in 73% yield over four steps (Scheme 23).

**Scheme 24.**
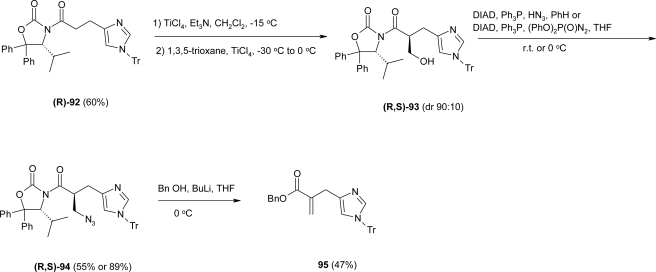
Diastereoselective aldol reaction of **(R)-92** with 1,3,5-trioxane. Further transformation towards the formation of β^2^-homo-histidine derivatives led to the formation of compound **95** [80].

*N*-acylation of the lithiated chiral auxiliary 4-isopropyl-5,5-diphenyl-1,3-oxazolidin-2-one (R)-DIOZ was accomplished by nucleophilic addition of the lithiated auxiliary (using BuLi in THF at 0 °C) to the pivaloyl mixed anhydride of **91** to give the acyloxazolidinone (R)-**92**, in 60% yield. The following aldol reaction was effected by reaction of the Ti-enolate of **92** with 1,3,5-trioxane to afford the hydroxymethyl derivative **93** in 70% yield. (Scheme 24).

**Scheme 25.**
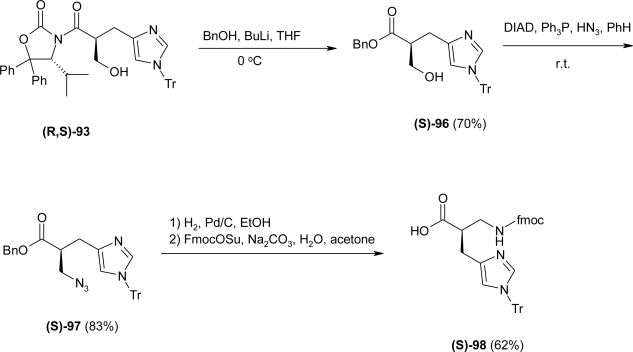
Removal of the auxiliary with BnOLi followed by Mitsunobu Reaction and functional group modifications for the preparation of Fmoc-(S)-β^2^hHis(Tr)-OH **98** [80].

The next step required the replacement of the OH group of **93** by a *N*-substituent by means of a Mitsunobu reaction. Thus, treatment of **(R**,**S)**-**93** with Ph_3_P, DIAD and either DPPA or hydrazoic acid afforded the azide derivative **(R**,**S)**-**94** in moderate to good yields (55 and 89% resp.). However attempts to cleave the auxiliary were not successful. Indeed, removal of the oxazolidinone group by BnOH/BuLi afforded the elimination product **95** (Scheme 24). To circumvent this problem, the hydroxymethyl derivative **93** was first treated with BnOH/BuLi to form the corresponding benzyl ester **(S)**-**96**, which was subsequently transformed to the azide **(S)**-**97** under the Mitsunobu conditions in good yield (Scheme 25).

Simultaneous benzyl ester cleavage and azide reduction, by catalytic hydrogenation followed by Fmoc-protection, afforded the histidine derivative **(S)-98** in moderate yield. It should be noted that the Tr protection group is stable under the hydrogenation conditions only to a certain extent; prolonged reaction times cause complete cleavage of that group.

In this way, the synthesis of (S)-Fmoc-β^2^hHis(Tr)-OH was achieved with an overall yield of 11% over ten steps from 4-isopropyl-5,5-diphenyl-1,3-oxazolidin-2-one (DIOZ).

### 4.10. β^3^-Homo-Histidine

**Scheme 26.**
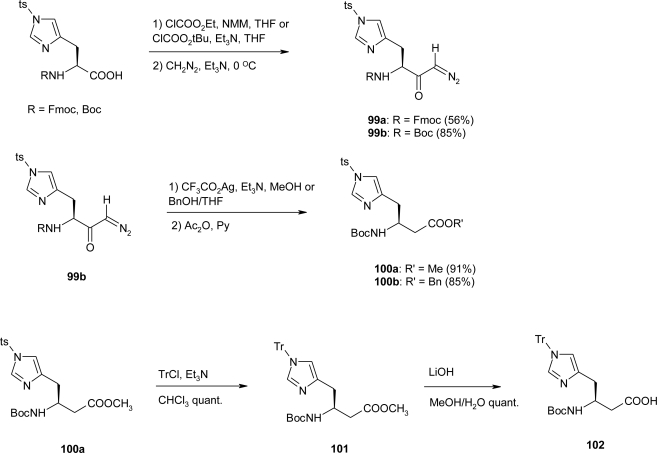
Preparation of the β^3^-homo-histidine derivative **102** by Arndt-Eistert homologation of Boc-His(Ts)-OH, followed by Tr-protection and saponification of the methyl ester group in **101** [80].

First attempts at synthesizing the homo-histidine derivatives by Seeback *et al*. [80] using the Arndt-Eistert homologation from Fmoc-His(τ-BOM)-OH, Fmoc-His(τ-Tr)-OH, Boc-His(τ-Bn)-OH, Boc-His(π-Bn)-OH, and Boc-His(τ-BOM)-OH were unsuccessful. Only Ts-protected histidines reacted with CH_2_N_2_ to form the corresponding diazo ketones, probably due to the strong electron-withdrawing effect of the tosylate, which renders the 1*H*-imidazolyl-*N*-atom less nucleophilic. Thus, commercially available Fmoc-His(Ts)-OH and Boc-His(Ts)-OH were converted via their mixed anhydrides (NMM/ClCO_2_Et or NEt_3_/ClCO_2_^i^Bu) to the diazo ketones **99a** and **99b** in 56 and 86% yields respectively (Scheme 26). Attempts at converting diazo ketone **99a** to a β^3^-homo-histidine derivative were shown to be ineffective, due to its insolubility in most solvents suitable for the Wolff rearrangement (e.g., BnOH, H_2_O, THF, dioxane, *etc*.). On the other hand, decomposition of diazo ketone **99b** in the presence of the corresponding alcohol (MeOH, BnOH), by reaction with catalytic amounts of Ag^+^ (CF_3_CO_2_Ag dissolved in Et_3_N) gave the Boc-protected methyl or benzyl ester **100** as a mixture of three products in a 1:1:1 ratio. It was found that the Ag^+^ ion interacts with the 1*H*-imidazole ring inducing the partial displacement of the Ts protecting group from the τ-N to the π-N, as well as the complete removal of this protecting group. With this result at hand, they decided to use Boc-His(πTs)-OH as starting material for conversion to the β^3^-amino acid derivative Fmoc-β^3^hHis(πTr)-OH. Since the use of histidine derivatives with unprotected 1*H*-imidazolyl side chains for peptide couplings in solution or on solid support are known to cause side reactions, Tr-protected 1*H*-imidazolyl group of the ester **100** was used: treatment with TrCl and Et_3_N afforded compound **101** in quantitative yield. Hydrolysis of the methyl ester with LiOH in MeOH/H_2_O gave the acid **102** as a single product (Scheme 26). The Boc-β^3^hHis(Tr)-OH **102** was thus prepared from α-Boc-His(Ts)-OH in ca. 75% yield over four steps.

**Scheme 27.**
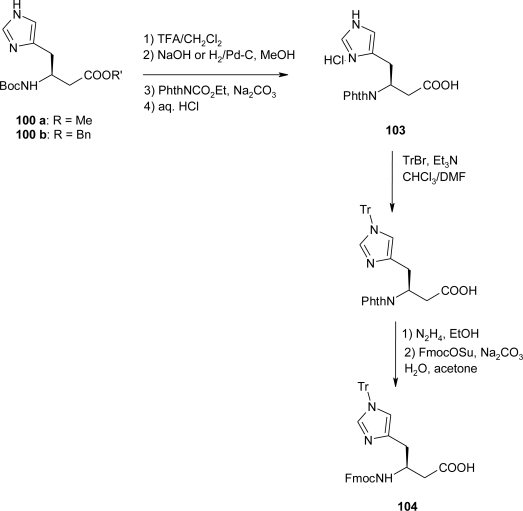
Preparation of Fmoc-β^3^hHis(Tr)-OH **104** starting from **100** [80].

The successful preparation of Fmoc-β^3^hHis(Tr)-OH was accomplished by using the Boc-protected homo-histidine esters **100** as starting materials. As such, Boc deprotection, followed by saponification or hydrogenolysis of the ester groups, gave the completely unprotected β^3^-homo-histidine, which was then phthaloyl(Phth)-protected and acidified to yield the HCl salt **103** in 67% yield. Subsequent tritylation and *N*-Phth/*N*-Fmoc protecting-group exchange afforded the acid **104** in 61% yield over the three steps (Scheme 27).

In this way, the synthesis of Fmoc-β^3^hHis(Tr)-OH was achieved in eight steps with an overall yield of 32%, starting from commercial Boc-His(Ts)-OH.

**Scheme 28.**
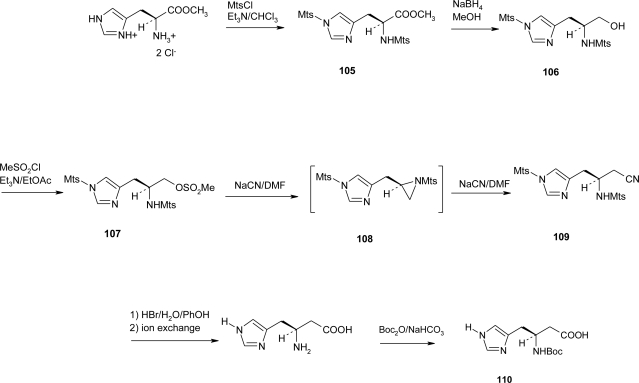
Preparation of Boc-β^3^hHis(Boc)-OH **110** via Kolbe reaction [81].

Recently, Wyatt and co-workers [81] accomplished the synthesis of Boc-β^3^hHis-(Boc)-OH **110** via the Kolbe reaction, *i.e*., (Scheme 28), by reducing α-Mts-His(Mts)-OMe **105** to the corresponding amino alcohol **106** in 58% yield, the OH group of which was then activated as its methanesulfonate **107** (81%) and replaced by CN, to give compound **109**; treatment of the methanesulfonate **107** with NaCN (1 equiv.) in DMF at room temperature gave mainly the aziridine **108**, which could be ring-opened by excess cyanide to give the desired nitrile **109**; by using two or more equivalents of NaCN in DMF at room temperature the methanesulfonate **107** could be converted into the nitrile **109** (63%) in a single step. The CN group was, in turn, hydrolyzed to the MeNH group, which was Boc-protected to give the final product **110** in 7% yield over five steps. The authors state that this derivative is suitable for direct use in the synthesis of peptides, but so far no applications are known.

### 4.11. Aza-Histidine

**Figure 10.**
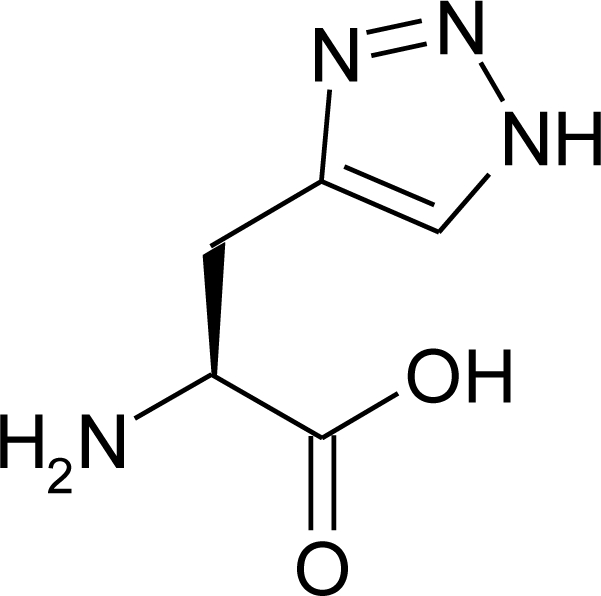
Triazole analogue of histidine.

The preparation of aza-histidine (Figure 10) has been already described [82] and the synthetic routes published to date to obtain the fully deprotected amino acid are usually rather long (seven steps) [83].

The recent development of the copper catalysed version of Huisgen cycloaddition allows the rapid access to 1,2,3-triazoles then aza-histidine derivatives have been obtained with this method, but no real attention has been paid to the removal of the protecting groups.

**Scheme 29.**
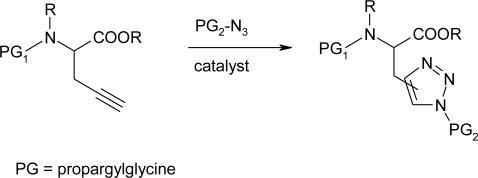
Aza-histidine analogues via [3 + 2] Huisgen cycloaddition [82].

Cintrat *et al*. [82] described the click chemistry based access to fully protected aza-histidine, suitable for solid phase peptide synthesis (Scheme 29).

Aza-histidine were obtained using either copper-catalysed click chemistry or the more recently described ruthenium catalyzed cycloaddition, affording the 1,5-disubstituted triazole. Nevertheless, the latter requires the fully protected propargylglycine as starting material, since the catalyst does not allow the use of free carboxylic acids. Using either condition, rapid access to protected aza-histidine was demonstrated and the expected regioisomers were obtained with fair to satisfactory yields.

**Scheme 30.**
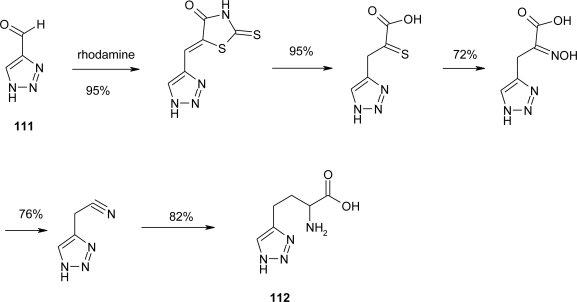
Synthesis of dl-α-Amino-1,2,3-triazole-4-propionic acid [84].

Robinson *et al*. [84] proposed the synthesis of dl-α-Amino-l,2,3-triazole-4-propionic acid **112**, prepared by two independent routes. The catalytic hydrogenation of the oximino acid was extremely slow and the yield of the amino acid was small (17%). As an unambiguous method, the synthesis was also accomplished through the azlactone. From 1,2,3-triazole-4-carboxaldehyde **111**, the crude azlactone was obtained (52%) as a mixture of a ring-acetylated azlactone and a small amount of the non-acetylated form which was separated through its insolubility in chloroform. The latter compound was readily converted into the former by acetylation. Purified samples of both forms gave α-benzamido-1,2,3-triazole-4-acrylic acid dihydrate on hydrolysis (86–88%). The yield of the acrylic acid dihydrate from the crude mixture of azlactones was 85%. α-Benzamido-l,2,3-triazole-4-propionic acid was obtained in 61% yield by hydrogenation of the acrylic acid in glacial acetic acid using platinum oxide. Hydrolysis of this benzamido acid gave analytically pure dl-amino-l,2,3-triazole-4-propionic acid **112** in 51% yield (Scheme 30).

**Scheme 31.**
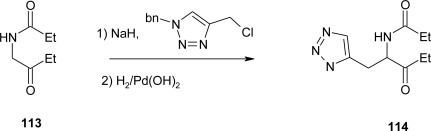
Synthesis of substituted aza-histidine [85].

Finally, Boyd *et al*. [85] explored variations in the imidazole portion of α-adrenoceptor agonists, synthesizing a series of compounds, among them substituted (+,−)-aza-histidine, shown in Scheme 31. The anion of ketoamide **113** was alkylated with a benzyl-protected heteroarylmethyl chloride to furnish the protected amidoketone **114**. Debenzylation of these compounds was accomplished by catalytic hydrogenation with Pearlman’s catalyst [Pd(OH)_2_]. Finally, the target compounds were obtained by cyclization of these intermediate to thiazole.

## 5. Conclusions

Topographical considerations are an important approach for exploring the stereochemical requirements for receptor recognition and for signal transduction [2,86].

In this approach, side chain constrained novel amino acids are designed and incorporated into peptide templates. The use of pure chiral α- or β-substituted amino acids in bioactive peptide ligands as key pharmacophore residues has proven to be a powerful tool for understanding ligand-receptors binding interaction and in peptidomimetics design [7].

**Figure 11.**
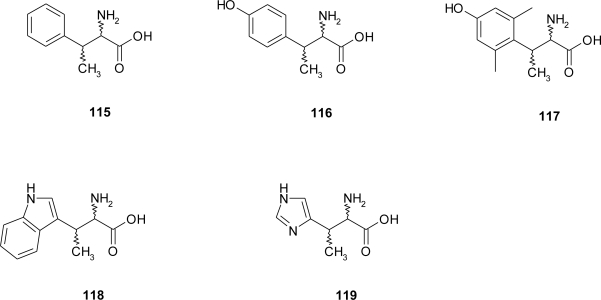
Some β-methyl-substituted amino acid analogues [87–91].

One of the major goals of medicinal chemistry has been the design and synthesis of novel amino acids with conformationally constrained side chains in order to obtain highly selective and potent peptide hormone and neurotransmitter analogues. Incorporation of different β-substituted amino acids (Figure 11), e.g., β-methylphenylalanine **115**, β-methyltyrosine **116**, β-methyl-2’,6’-dimethyltyrosine **117**, and β-methyltryptophan **118**, provided new insights into the stereochemical requirements of peptide-receptor interactions [87–90]. Beta-methylhistidine (**119** in Figure 11) is an important amino acid involved in signal transduction mechanism necessary to express biological activities [91] and in several key role receptor interactions, one of which being glucagone with its receptor.

Topographical changes can greatly affect the potency and selectivity of peptidic ligands. Though this approach to ligand design is embryonal, significant progress has been made and some very impressive peptide-based ligands have been discovered.

The major difficulties to synthesize substituted asymmetric histidine derivatives arise from the imidazole ring, which is a general base and a nucleophile. Thus, the imidazole group can cause epimerization and may participate in different reactions during the synthesis of asymmetric amino acid derivatives [8].

There remains a need for a deeper understanding of the conformational properties of many of these amino acids as well as for the design, synthesis and conformational analysis of novel amino acids with well-defined χ^1^ and χ^2^ angles.

### The Importance of Histidine Amino Acid as Residue in Bioactive Molecules and in the Synthesis of Biologically Active Compounds

**Figure 12.**
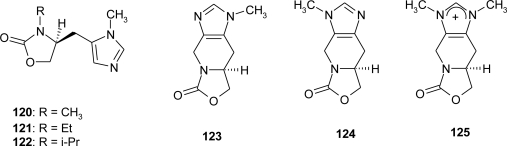
(+) Pilocarpine analogs [92].

An example of the use of l-histidine in the synthesis of biologically active compounds is reported by Gonzales *et al*. [92]: They prepared the cyclic carbamate analogs of **120**–**122** as a part of a novel series of muscarinic agonists containing the 2-oxazolidinone ring system, and the conformationally restricted derivatives **123**–**125** (Figure 12), the latter synthesized by the Pictet-Spengler reaction.

It is of interest that no rigid analog of (+)-pilocarpine has been reported to have more than approximately 1% of the agonist activity of the naturally occurring alkaloid.

**Figure 13.**
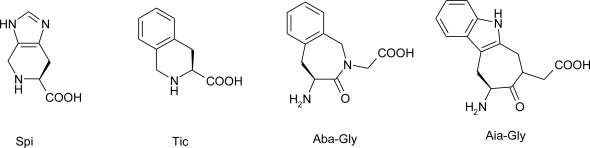
Conformationally constrained aromatic residues used as His replacements in Ang IV [93].

Tourwè *et al*. [93] replaced the histidine residue in angiotensin IV by various conformationally constrained amino acids (Figure 13).

The substitution of the His^4^-Pro^5^ dipeptide sequence by the constrained Trp analogue Aia-Gly, in combination with β^2^hVal substitution at the *N*-terminus, provided a new stable analogue H-(R)-β^2^hVal-Tyr-Ile-Aia-Gly-Phe-OH (AL-40) that is a potent ligand for the Ang IV receptor IRAP and selective *versus* AP-N and the AT1 receptor.
